# Aging differentially modulates the Wnt pro‐survival signalling pathways in vascular smooth muscle cells

**DOI:** 10.1111/acel.12844

**Published:** 2018-12-12

**Authors:** Bethan A. Brown, Georgia M. Connolly, Carina E. J. Mill, Helen Williams, Gianni D. Angelini, Jason L. Johnson, Sarah J. George

**Affiliations:** ^1^ Bristol Medical School University of Bristol, Bristol Royal Infirmary Bristol UK

**Keywords:** aging, apoptosis, atherosclerosis, oxidative stress, vascular smooth muscle cell, Wnt

## Abstract

We previously reported pro‐survival effects of Wnt3a and Wnt5a proteins in vascular smooth muscle cells (VSMCs). Wnt5a achieved this through induction of Wnt1‐inducible signalling pathway protein‐1 (WISP‐1) consequent to β‐catenin/CREB‐dependent, TCF‐independent, signalling. However, we found that as atherosclerosis advances, although Wnt5a protein was increased, WISP‐1 was reduced. We hypothesized this disconnect could be due to aging. In this study, we elucidate the mechanism underlying Wnt3a pro‐survival signalling and demonstrate the differential effect of age on Wnt3a‐ and Wnt5a‐mediated survival. We show Wnt3a protein was expressed in human atherosclerotic coronary arteries and co‐located with macrophages and VSMCs. Meanwhile, Wnt3a stimulation of primary mouse VSMCs increased β‐catenin nuclear translocation and TCF, but not CREB, activation. Wnt3a increased mRNA expression of the pro‐survival factor WISP‐2 in a TCF‐dependent manner. Functionally, β‐catenin/TCF inhibition or WISP‐2 neutralization significantly impaired Wnt3a‐mediated VSMC survival. WISP‐2 was upregulated in human atherosclerosis and partly co‐localized with Wnt3a. The pro‐survival action of Wnt3a was effective in VSMCs from young (2 month) and old (18–20 month) mice, whereas Wnt5a‐mediated rescue was impaired with age. Further investigation revealed that although Wnt5a induced β‐catenin nuclear translocation in VSMCs from both ages, CREB phosphorylation and WISP‐1 upregulation did not occur in old VSMCs. Unlike Wnt5a, pro‐survival Wnt3a signalling involves β‐catenin/TCF and WISP‐2. While Wnt3a‐mediated survival was unchanged with age, Wnt5a‐mediated survival was lost due to impaired CREB activation and WISP‐1 regulation. Greater understanding of the effect of age on Wnt signalling may identify targets to promote VSMC survival in elderly patients with atherosclerosis.

## 1 INTRODUCTION

Age is an established risk factor for cardiovascular disease (reviewed in Wang & Bennett, 2012), which despite extensive research, remains the leading cause of death worldwide (WHO, 2011). The basis for prevalent cardiovascular pathologies, such as heart attacks and strokes, is a progressive inflammatory disease of the vasculature termed atherosclerosis. Atherosclerosis progresses over years (Virmani et al., 1991), eventually culminating in the development of advanced plaques consisting of a lipid‐rich inflammatory core covered with a protective fibrous cap containing vascular smooth muscle cells (VSMCs) and collagenous extracellular matrix (Bennett, Sinha, & Owens, 2016; Libby, 2012). Death of VSMCs within the fibrous cap promotes cap thinning and increases the likelihood of plaque rupture, thrombosis and ischaemia (von der Thusen et al., 2002, Clarke et al., 2006). Thus, blockade of VSMC apoptosis represents an intriguing target for therapeutic intervention to inhibit plaque instability and associated mortality.

Accumulating evidence suggests that β‐catenin‐dependent (canonical) signalling by Wnt proteins is implicated in the regulation of VSMC survival (Hall, Chatham, Eldar‐Finkelman, & Gibbons, 2001; Mill et al., 2014; Wang et al., 2002). The Wnt family consists of 19 cysteine‐rich secreted glycoproteins (https://web.stanford.edu/group/nusselab/cgi-bin/wnt/) which bind to frizzled receptors and low‐density lipoprotein receptor‐related protein‐5/6 (LRP5/6) co‐receptors on the cell surface. This causes inhibition of the β‐catenin destruction complex, which contains casein kinase‐1α, glycogen synthase kinase‐3β, AXIN and adenomatous polyposis coli. This permits β‐catenin accumulation, nuclear translocation and binding of other transcription factors such as members of the T‐cell factor (TCF)/lymphoid enhancer‐binding factor family to modify gene transcription (Clevers & Nusse, 2012). Activation of β‐catenin has been observed in human carotid atherosclerotic lesions (Bedel et al., 2008); however to date, only one Wnt member, Wnt5a, has been directly investigated in atherosclerosis (Christman et al., 2008; Mill, Tsaousi, Woodward, Johnson, & George, 2010).

A previous study by our group identified a pro‐survival effect of Wnt5a in primary murine VSMCs challenged with hydrogen peroxide (H_2_O_2_; Mill et al., 2014)*.* Further investigation revealed that although Wnt5a protein alone induced β‐catenin nuclear translocation and β‐catenin/TCF reporter activation, in the presence of H_2_O_2,_ while β‐catenin nuclear translocation was maintained, and β‐catenin/TCF reporter activation was lost (Mill et al., 2014). Similarly, Wnt5a upregulated the β‐catenin/TCF‐responsive genes survivin, insulin‐like growth factor‐1 (IGF‐1) and WNT1‐inducible signalling pathway protein‐2 (WISP‐2) alone, but not in the presence of H_2_O_2_. Hence, we concluded that as TCF signalling was inhibited by the presence of oxidative stress, this mechanism was not involved in pro‐survival Wnt5a signalling (Mill et al., 2014). Instead, we showed that Wnt5a‐mediated suppression of apoptosis was dependent on CREB activation and upregulation of WISP‐1 (Mill et al., 2014). Furthermore, we also reported that another Wnt, Wnt3a, rescued VSMCs from H_2_O_2_‐induced apoptosis and upregulated WISP‐1 mRNA (Mill et al., 2014); however, the signalling pathway involved in this pro‐survival effect was not investigated further.

Immunohistochemistry by our group revealed that Wnt5a and WISP‐1 proteins were both present in human coronary atherosclerotic lesions; however, WISP‐1 protein did not co‐localize to Wnt5a‐positive VSMCs (Mill, Jeremy, & George, 2011). In addition, although Wnt5a increased with plaque instability, WISP‐1 levels were reduced and were associated with enhanced VSMC apoptosis in the fibrous cap (Mill et al., 2014, 2010 ). These data imply that in advanced disease, Wnt5a‐mediated WISP‐1 expression and VSMC survival may be impaired. As aging is a risk factor for atherosclerosis (Wang & Bennett, 2012), it was hypothesized that failure of Wnt5a to promote VSMC survival and WISP‐1 expression in advanced atherosclerosis may be due to aging.

In this study, we investigated the mechanism of pro‐survival signalling activated by Wnt3a in VSMCs, and whether either Wnt3a‐ or Wnt5a‐mediated rescue of oxidative stress‐induced apoptosis was affected by aging. To achieve this, murine VSMCs were employed, as previous reports suggest that mouse VSMCs may represent a more translatable model for human VSMC aging compared to rat VSMCs (reviewed by Orlandi, Bochaton‐Piallat, Gabbiani, & Spagnoli, 2006, Monk & George, 2014, discussed by Moon et al., 2001, Rodriguez‐Menocal et al., 2010).

## 2 RESULTS

### 2.1 Wnt3a was expressed in human atherosclerotic lesions

**Figure 1:**
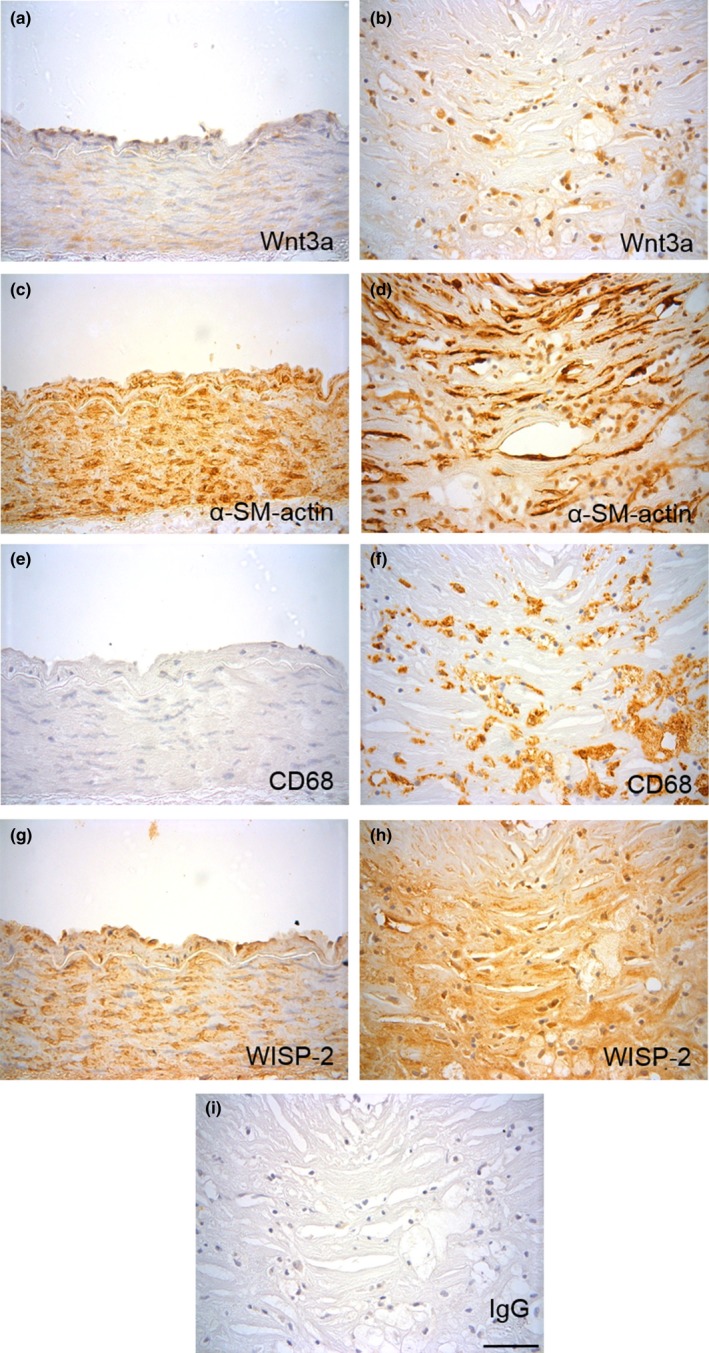
Wnt3a and WISP‐2 proteins are upregulated in human atherosclerotic plaques. Representative images of immunohistochemistry for Wnt3a (a, b), α‐smooth muscle actin (c, d), CD68 (e, f) and WISP‐2 proteins (g, h) in control (a, c, e & g) and atherosclerotic (b, d, f & h) human coronary arteries. Non‐immune rabbit IgG was included as a negative control for Wnt3a and WISP‐2 antibodies (i). The scale bar represents 50 μm and applies to all images. A lower magnification image of each vessel is available in Supporting Information Figure S1

To assess Wnt3a protein expression in human coronary artery atherosclerotic lesions, immunohistochemistry was performed (Figure 1a,b and Supporting Information Figure S1). Wnt3a protein was barely detected in non‐diseased coronary arteries but was significantly upregulated in atherosclerotic plaques (2.11 ± 0.35‐fold, *n* = 9 plaque and *n* = 4 non‐diseased, *p* < 0.05). Adjacent to the plaque core, Wnt3a protein was observed in areas containing α‐smooth muscle actin‐positive VSMCs and CD68‐positive macrophages (Figure 1c–f). These results demonstrate that Wnt3a is present in human atherosclerosis and may affect VSMC behaviour in the plaque.

### 2.2 Wnt3a activated β‐catenin and TCF but not CREB

**Figure 2:**
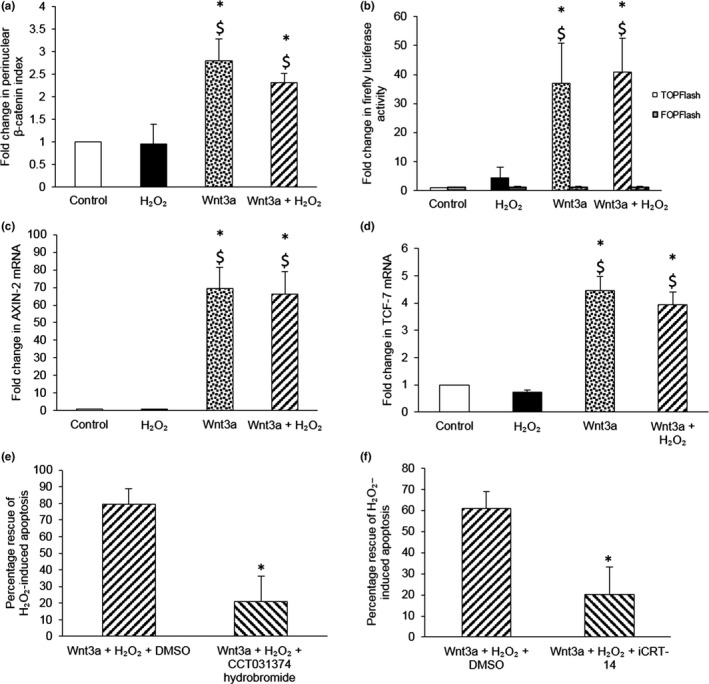
Wnt3a‐mediated rescue of VSMCs from H_2_O_2_‐induced apoptosis requires β‐catenin/TCF signalling. (a) Nuclear translocation of β‐catenin was quantified in young TOPGAL mouse VSMCs stimulated with 100 μM H_2_O_2_, with or without 400 ng/ml recombinant Wnt3a protein, for 30 min by immunofluorescence. The number of cells with β‐catenin nuclear translocation, defined as perinuclear β‐catenin staining, was counted and expressed as a percentage of the total number of cells viewed. The fold change in the percentage of cells with perinuclear β‐catenin vs. control was then calculated and referred to as the fold change in perinuclear β‐catenin index. Error bars represent *SEM*. *p < 0.05 vs. control, $*p* < 0.05 vs. H_2_O_2_, ANOVA and Student–Newman–Keuls post hoc test, *n* = 4. (b) Firefly luciferase activity was quantified in young TOPGAL mouse VSMCs transfected with TOPFlash or FOPFlash plasmids (negative control) and stimulated with 100 μM H_2_O_2_, with or without 400 ng/ml recombinant Wnt3a protein, for 24 hr. Renilla luciferase plasmids were used to normalize for transfection efficacy, prior to normalization by protein concentration. Results are shown as the fold change from control. Error bars represent *SEM*. *p < 0.05 vs. control, $*p* < 0.05 vs. H_2_O_2_, ANOVA and Student–Newman–Keuls post hoc test, *n* = 6. (c, d) *AXIN‐2* (c) or *TCF‐7* mRNA (d) was quantified by QPCR in young TOPGAL mouse VSMCs stimulated with 100 μM H_2_O_2_, with or without 400 ng/ml recombinant Wnt3a protein, for 4 hr. mRNA levels were normalized to *36B4* mRNA. Results are shown as the fold change from control. Error bars represent *SEM*. *p < 0.05 vs. control, $*p* < 0.05 vs. H_2_O_2_, ANOVA and Student–Newman–Keuls post hoc test, *n* = 3. (e, f) Apoptosis was quantified in young TOPGAL mouse VSMCs stimulated with 100 μM H_2_O_2_, with or without 400 ng/ml recombinant Wnt3a protein and either 1 μM CCT031374 hydrobromide (e) or 25 μM iCRT14 (f), for 24 hr using CC3 immunofluorescence. DMSO was used as a vehicle control. The number of CC3 positive cells was counted and expressed as a percentage of the total number of cells viewed. The percentage rescue from H_2_O_2_‐induced death was then calculated. Error bars represent *SEM*. *p < 0.05 vs. the percentage rescue by Wnt3a protein in the presence of DMSO, unpaired Student’s *t* test, *n* = 4 for (e) and *n* = 5 for (f)

We previously reported that recombinant Wnt3a protein significantly suppressed H_2_O_2_‐induced VSMC apoptosis (Mill et al., 2014). To delineate the signalling pathway involved, the ability of Wnt3a to activate β‐catenin, TCF and CREB was analysed. Wnt3a, in both the absence and presence of H_2_O_2_, significantly increased the percentage of cells with perinuclear β‐catenin (Figure 2a and Supporting Information Figure S2). In addition, Wnt3a, with and without H_2_O_2_, significantly induced β‐catenin/TCF‐mediated TOPFlash luciferase expression (Figure 2b) and mRNA levels of β‐catenin/TCF‐responsive genes *AXIN‐2* (Jho et al., 2002) and *TCF‐7* (Roose et al., 1999; Figure 2c–d). However, Wnt3a did not increase the levels of active phosphorylated CREB (ser133; Supporting Information Figure S3).

To test whether β‐catenin/TCF signalling was necessary for Wnt3a‐mediated survival, VSMCs were treated with β‐catenin/TCF inhibitors: CCT031374 hydrobromide (Ewan et al., 2010) and iCRT‐14 (Gonsalves et al., 2011). In DMSO vehicle controls, Wnt3a protein significantly rescued H_2_O_2_‐induced apoptosis. However, in the presence of either inhibitor, rescue was significantly impaired (Figure 2e,f). Of note, neither inhibitor significantly increased basal or H_2_O_2_‐induced apoptosis (data not shown, *n* = 4–5).

### 2.3 WISP‐2 was essential for Wnt3a‐mediated survival

**Figure 3:**
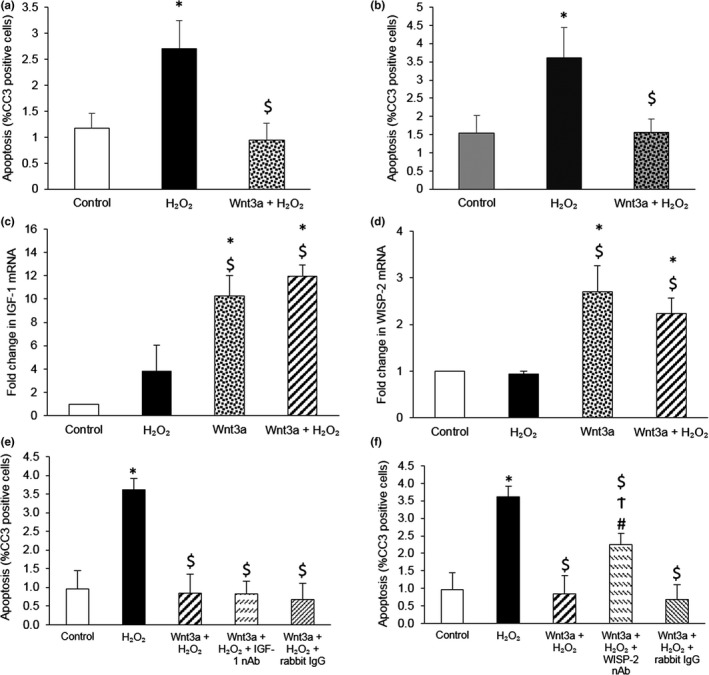
Wnt3a upregulated multiple pro‐survival genes, but only WISP‐2 was necessary for Wnt3a‐mediated rescue of VSMCs from H_2_O_2_‐induced apoptosis. (a, b) Apoptosis was quantified in young TOPGAL mouse VSMCs transfected with either AllStars Negative Control siRNA (a) or WISP‐1 siRNA (b), and then 24 hr later stimulated with 100 μM H_2_O_2_, with or without 400 ng/ml recombinant Wnt3a protein, for a further 24 hr. Apoptosis was quantified using CC3 immunofluorescence. The number of CC3 positive cells was counted and expressed as a percentage of the total number of cells viewed. Error bars represent *SEM*. *p < 0.05 vs. control, $*p* < 0.05 vs. H_2_O_2,_ repeated measures ANOVA and Student–Newman–Keuls post hoc test_,_ *n* = 5. (c, d) *IGF‐1* (c) and *WISP‐2* (d) mRNAs were quantified by QPCR in young TOPGAL mouse VSMCs stimulated with 100 μM H_2_O_2_, with or without 400 ng/ml recombinant Wnt3a protein, for 4 hr. mRNA levels were normalized to *36B4* mRNA levels. Results are shown as the fold change from control. Error bars represent *SEM*. *p < 0.05 vs. control, $*p* < 0.05 vs. H_2_O_2_, ANOVA and Student–Newman–Keuls post hoc test, *n* = 3. (e, f) Apoptosis was quantified in young TOPGAL mouse VSMCs stimulated with 100 µM H_2_O_2_, with or without 400 ng/ml recombinant Wnt3a protein and 10 µg/ml IGF‐1 neutralizing antibody (nAb) (e) or 10 µg/ml WISP‐2 neutralizing antibody (nAb) (f), for 24 hr using CC3 immunofluorescence. Non‐immune rabbit IgG acted as a negative control. The number of CC3 positive cells was counted and expressed as a percentage of the total number of cells viewed. Error bars represent *SEM*. *p < 0.05 vs. control, $*p* < 0.05 vs. H_2_O_2_, #*p* < 0.05 vs. Wnt3a + H_2_O_2_, Ϯ*p* < 0.05 vs. Wnt3a + H_2_O_2_ + rabbit IgG, repeated measures ANOVA and Student–Newman–Keuls post hoc test, *n* = 5

We previously found that Wnt3a upregulated WISP‐1 expression in murine VSMCs (Mill et al., 2014). To determine whether WISP‐1 was necessary for Wnt3a‐mediated survival, knockdown of WISP‐1 was performed using our previously validated protocol (Mill et al., 2014). Surprisingly, WISP‐1 knockdown did not significantly impair Wnt3a‐mediated rescue (Figure 3a,b). To identify the genes involved in Wnt3a‐mediated survival, mRNA levels of three other β‐catenin/TCF‐responsive pro‐survival genes, *survivin, IGF‐1* and *WISP‐2,* were investigated (Bennett, Evan, & Schwartz, 1995; Longo et al., 2002; Mill et al., 2014; Ohkawa et al., 2011; Wang et al., 2005; Zhang et al., 2001). *Survivin* mRNA was not affected by Wnt3a (data not shown, *n* = 3); however, *IGF‐1* and *WISP‐2* mRNAs were upregulated by Wnt3a alone and with H_2_O_2_ (Figure 3c,d).

To investigate the role of IGF‐1 and WISP‐2 in Wnt3a‐mediated survival, neutralizing antibodies that significantly retarded the suppression of apoptosis by recombinant IGF‐1 and WISP‐2 proteins (Supporting Information Figure S4) were utilized. Neutralization of WISP‐2, but not IGF‐1, significantly impaired Wnt3a‐mediated rescue of H_2_O_2_‐induced apoptosis (Figure 3e,f). Furthermore, WISP‐2 mRNA upregulation by Wnt3a was inhibited by iCRT‐14 (46.1% ± 15.0% inhibition of WISP‐2 induction by Wnt3a, 38.0% ± 14.7% inhibition of WISP‐2 induction by Wnt3a + H_2_O_2_, one‐sample *t* test compared to 0% inhibition, *p* < 0.05, *n* = 6), establishing a direct connection between TCF and WISP‐2 following Wnt3a treatment.

### 2.4 WISP‐2 is expressed in human atherosclerosis

Similar to Wnt3a, WISP‐2 protein was also upregulated in atherosclerosis compared to non‐diseased arteries (2.64 ± 0.2‐fold, *n* = 11 plaque and *n* = 4 non‐diseased, *p* < 0.05) (Figure 1g,h and Supporting Information Figure S1).

### 2.5 Divergent Wnt3a and Wnt5a signalling was not due to differential Fzd binding

Microarrays performed by Tsaousi *et al*. found that only two Fzds, Fzd1 and Fzd6, were consistently expressed at detectable levels in murine aortic VSMCs (Tsaousi et al., 2011). To determine whether the divergent pathways activated by Wnt3a and Wnt5a were due to differential binding to Fzd1 or Fzd6, siRNA‐mediated knockdown of each receptor was performed. Supporting Information Figure S5 shows that in the absence of either receptor, Wnt3a‐mediated rescue was maintained, whereas Wnt5a‐mediated survival was lost. These data imply that Wnt5a requires the presence of both receptors to inhibit apoptosis, whereas Wnt3a signalling is maintained as long as either receptor is present. Importantly, these results demonstrate that differential use of Fzd1 and Fzd6 does not underlie the divergent pathways activated by these two Wnts.

### 2.6 The divergent Wnt3a and Wnt5a pathways were differentially affected by aging

**Figure 4:**
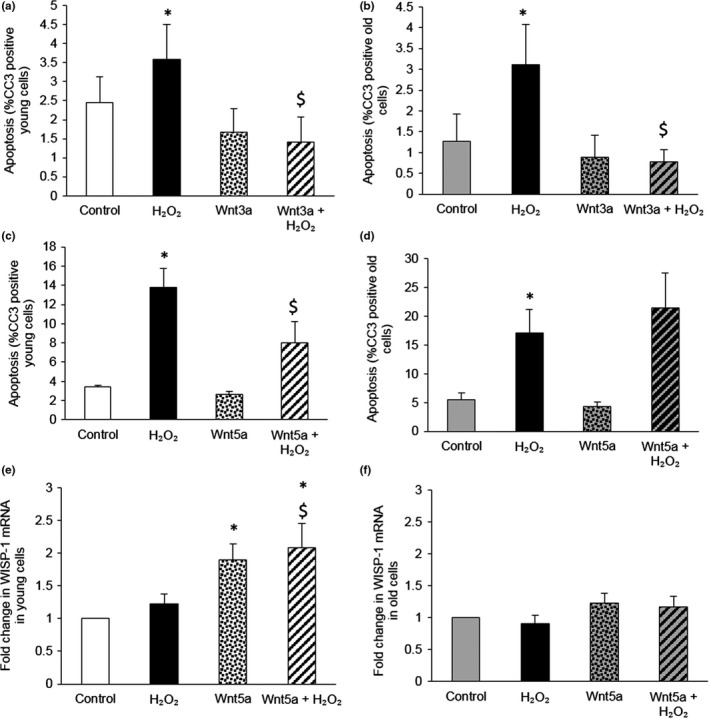
Wnt3a protein inhibited H_2_O_2_‐induced apoptosis in VSMCs from young and old mice, whereas Wnt5a‐mediated rescue and WISP‐1 upregulation was lost with age. (a–d) Apoptosis was quantified in VSMCs isolated from young (a, c) and old (b, d) mice and stimulated with 100 μM H_2_O_2_, with or without 400 ng/ml recombinant Wnt3a (a, b) or Wnt5a protein (c, d), for 24 hr using CC3 immunofluorescence. The number of CC3 positive cells was counted and expressed as a percentage of total number of cells viewed. *p < 0.05 vs. control, $*p* < 0.05 vs. H_2_O_2_, repeated measures ANOVA and Student–Newman‐Keuls post hoc test, *n* = 3 for C and *n* = 5 for (a, b and d). (e, f) *WISP‐1* mRNA was quantified by QPCR in VSMCs isolated from young (e) and old (f) mice and stimulated with 100 μM H_2_O_2_, with or without 400 ng/ml recombinant Wnt5a protein, for 4 hr. *WISP‐1* mRNA levels were quantified from a standard curve. Results are shown as the fold change from control. Error bars represent mean ± *SEM*. *p < 0.05 vs. control, $*p* < 0.05 vs. H_2_O_2_, ANOVA and Student–Newman–Keuls post hoc test, *n* = 7 for (e) and *n* = 5 for (f)

Both Wnt3a and Wnt5a recombinant proteins significantly inhibited H_2_O_2_‐induced apoptosis of VSMCs from young (2 month) mice; however, only Wnt3a rescued VSMCs from old (18–20 month) mice (Figure 4a–d, Supporting Information Figure S6). Moreover, although Wnt5a protein increased *WISP‐1* mRNA in young VSMCs, this response was absent in VSMCs from old mice (Figure 4e,f).

### 2.7 LRP5, LRP6 and Dkk3 mRNA, but not protein, were altered with aging

Initially, it was hypothesized that altered expression of Wnt signalling components with age could be responsible for the impaired Wnt5a‐mediated rescue observed in old VSMCs. As shown in Supporting Information Table S3, the mRNA level for the majority of Wnt signalling components did not differ with age. However, *Wnt2, Wnt8a, NCAD, LRP5* and *LRP6* mRNAs were significantly reduced in old VSMCs, whereas, *Dkk3* mRNA was significantly increased. Following this, further experiments were limited to investigate signalling components which were deemed most likely to inhibit Wnt5a signalling with age: low expression of the co‐receptors LRP5 and LRP6 (Mill et al., 2014) and high expression of the canonical Wnt inhibitor Dkk3 (Nakamura, Hunter, Yi, Brunken, & Hackam, 2007). However, western blotting of VSMC lysates revealed that age had no effect on LRP6 or Dkk3 proteins (Supporting Information Figure S7), and LRP5 protein was not detected.

### 2.8 Wnt5a‐mediated activation of CREB, but not β‐catenin, was impaired with age

**Figure 5:**
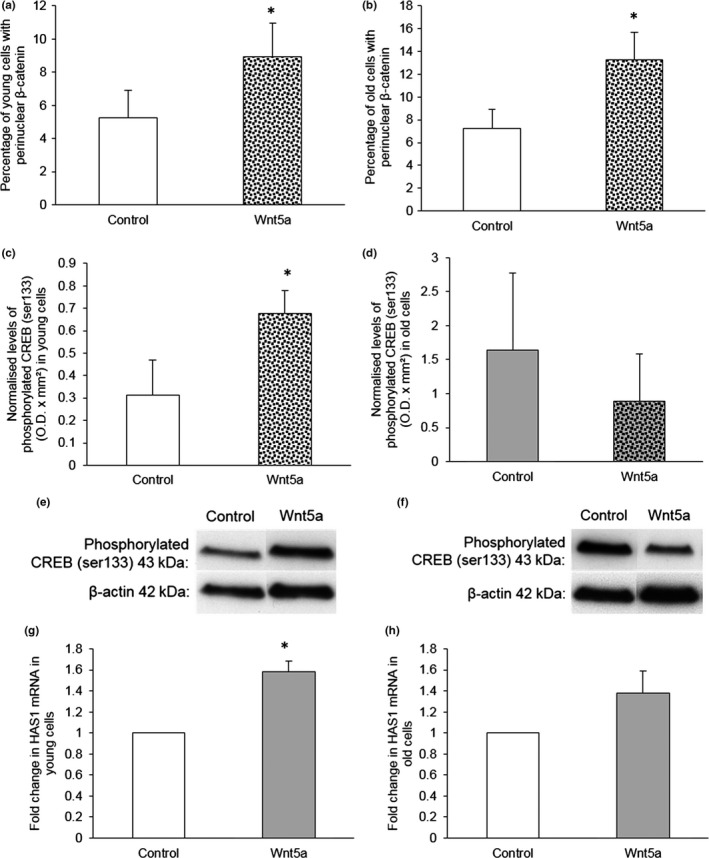
Wnt5a‐mediated activation of CREB, but not β‐catenin, was impaired by age. (a, b) β‐catenin nuclear translocation was quantified in VSMCs isolated from young (a) and old (b) mice and stimulated with 400 ng/ml recombinant Wnt5a protein for 30 min by immunofluorescence. The number of cells with β‐catenin nuclear translocation, defined as perinuclear β‐catenin staining, was counted and expressed as a percentage of the total number of cells viewed. Error bars represent mean ± *SEM*. *p < 0.05 vs. control, paired Student’s *t* test, *n* = 6 for (a) and *n* = 5 for (b). (c–f) Phosphorylated CREB (ser133) was detected by western blotting of VSMCs from young (c) and old (d) mice and stimulated with 400 ng/ml recombinant Wnt5a protein for 10 min. Levels of phosphorylated CREB (ser133) were normalized to β‐actin. *p < 0.05 vs. control, paired Student’s *t* test, *n* = 3. Representative western blots are shown (e, young and f, old). (g, h)* HAS1* mRNA was quantified by QPCR in VSMCs isolated from young (g) and old (h) mice and stimulated with 400 ng/ml recombinant Wnt5a protein for 2 hr. *HAS1* mRNA levels were normalized to *36B4* mRNA levels. Results are shown as the fold change from control. *p < 0.05 vs. control, one‐sample *t* test, *n* = 3 young and *n* = 4 old

To determine whether Wnt5a‐mediated activation of β‐catenin was affected by age, immunofluorescence to analyse β‐catenin nuclear translocation was performed. Wnt5a significantly increased the proportion of cells with perinuclear β‐catenin in both young and old VSMCs (Figure 5a,b and Supporting Information Figure S8). In addition, we found that mRNAs for the β‐catenin/TCF‐responsive genes *AXIN‐2* and *TCF‐7* were regulated in a similar manner by Wnt5a in young and old VSMCs (Supporting Information Figure S9).

On the other hand, the ability of Wnt5a to increase phosphorylated CREB (ser133) was only observed in VSMCs from young, not old, mice (Figure 5c–f). No significant difference in the basal levels of phosphorylated CREB was observed between VSMCs from young and old mice (0.31 ± 0.15 normalized young O.D. x mm^2^ vs. 1.63 ± 1.14 normalized old O.D. x mm^2^, unpaired Student’s *t* test with Welch correction, *p* > 0.05, *n* = 3). Wnt5a‐mediated CREB activation was also assessed by quantification of the CREB‐responsive gene *HAS1*. Wnt5a significantly induced *HAS1* expression in VSMCs from young, but not old, mice (Figure 5g,h) supporting the suggestion that Wnt5a‐mediated CREB activation was impaired with age and this may therefore be the cause of the diminished Wnt5a survival and WISP‐1 upregulation in old VSMCs.

## 3 DISCUSSION

### 3.1 Wnt3a‐mediated rescue of VSMC apoptosis involved β‐catenin/TCF but not CREB

**Figure 6:**
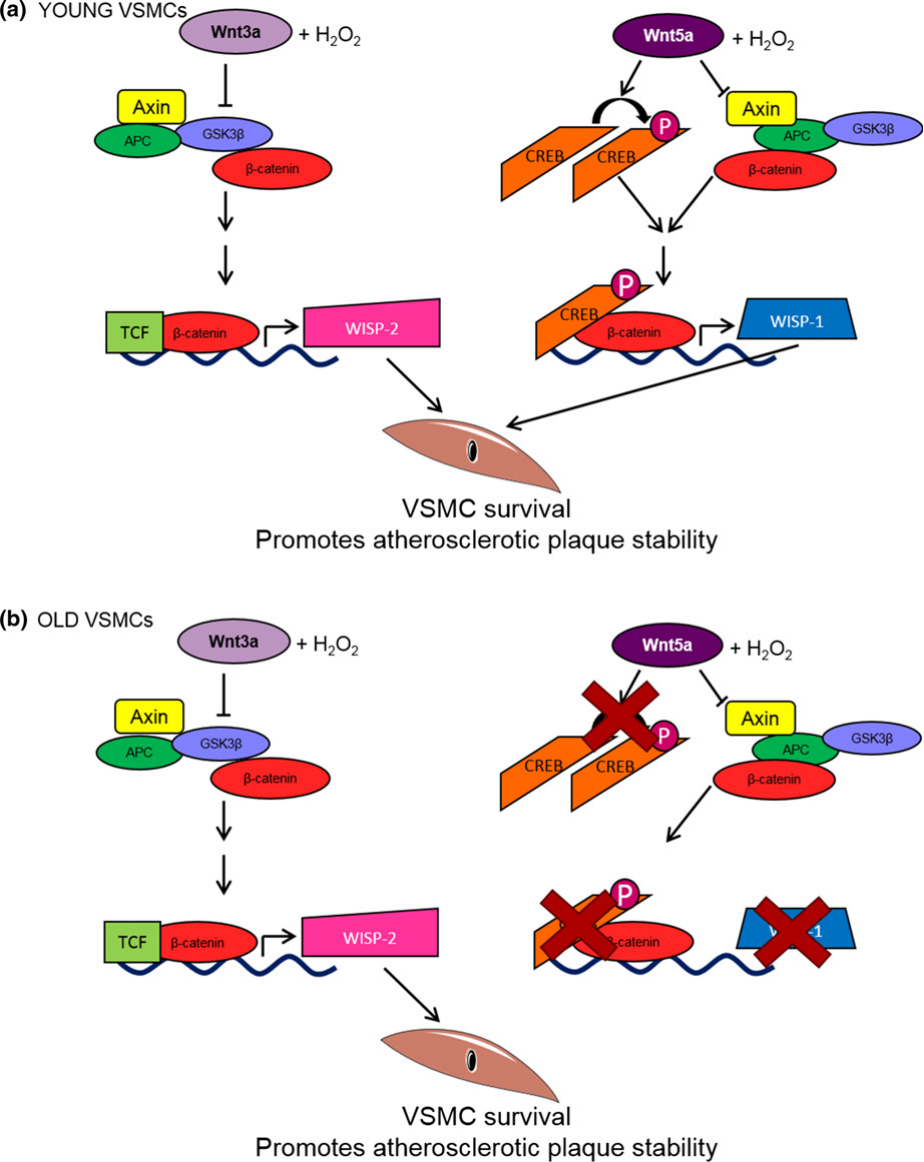
Wnt3a and Wnt5a signalling via divergent pathways to promote VSMC survival, and these pathways are differentially affected by aging. (a) In VSMCs from young mice, Wnt3a, in the presence of H_2_O_2_, activated β‐catenin/TCF‐mediated transcription and upregulation of the survival genes IGF‐1, WISP‐1 and WISP‐2. However, only WISP‐2 was necessary for rescue of H_2_O_2_‐induced VSMC apoptosis. Conversely, Wnt5a, in the presence of H_2_O_2_, induced β‐catenin nuclear translocation, but not β‐catenin/TCF activation. Instead, Wnt5a‐mediated rescue of VSMCs was dependent on CREB activation and CREB‐dependent WISP‐1 upregulation (Mill et al., 2014). Overall in young VSMCs, both Wnt pathways contribute to VSMC survival, which in atherosclerosis would promote fibrous cap maintenance and plaque stability. (b) In VSMCs from old mice, Wnt3a rescued H_2_O_2_‐induced apoptosis, whereas the ability of Wnt5a to phosphorylate CREB was impaired resulting in a lack of CREB‐dependent WISP‐1 upregulation and cell survival. Thus, in aged VSMCs, only one of the two Wnt pathways investigated here promotes VSMC survival. In atherosclerosis, this could impair fibrous cap maintenance predisposing fibrous cap thinning and plaque instability

This paper revealed that in contrast to Wnt5a, an alternative signalling pathway is involved in the pro‐survival effect of Wnt3a (Figure 6). We previously reported that Wnt5a‐mediated activation of β‐catenin/TCF signalling was inhibited by H_2_O_2_, and instead, WISP‐1 upregulation and suppression of apoptosis were dependent on CREB activation (Mill et al., 2014). However, in the current paper, we report that Wnt3a‐mediated activation of β‐catenin nuclear translocation and β‐catenin/TCF‐mediated gene transcription was maintained in the presence of H_2_O_2_. This implies that oxidative stress did not inhibit β‐catenin/TCF signalling by Wnt3a. Additionally, TCF was necessary for Wnt3a‐mediated VSMC survival as this effect was impaired by TCF inhibition.

Unlike Wnt5a (Mill et al., 2014), Wnt3a did not activate CREB. As we previously reported induction of WISP‐1 by Wnt3a (Mill et al., 2014), this finding was unexpected as activation of a human *WISP‐1* promoter by stable β‐catenin has previously been shown to require CREB, but not TCF/LEF‐binding sites (Xu, Corcoran, Welsh, Pennica, & Levine, 2000). That said, others have reported that in human VSMCs, activation of a *WISP‐1* reporter by IL‐18 could be inhibited by deletion of TCF sites (Reddy, Valente, Delafontaine, & Chandrasekar, 2011), while Wang *et al*. showed in human colonic epithelial cells, *WISP‐1* reporter induction by nitric oxide could be inhibited by β‐catenin siRNA or mutation of TCF or CREB promoter binding sites, suggesting the involvement of all three transcription factors in WISP‐1 expression (Wang et al., 2009).

### 3.2 WISP‐2 was necessary for Wnt3a‐mediated rescue of VSMC apoptosis

To our surprise, WISP‐1 knockdown had no significant effect on Wnt3a‐mediated survival. Together with findings by Mill et al. (2014), this suggests that although WISP‐1 is upregulated by both Wnt3a and Wnt5a, it is only necessary for the anti‐apoptotic effect of the latter. The reason for this discrepancy is unclear, but as splice variants of WISP‐1 have been reported to have differing effects on cell behaviour (Tanaka et al., 2001; Yanagita et al., 2007), further investigations could determine which WISP‐1 variants are involved in Wnt3a‐ and Wnt5a‐mediated survival.

We report that the β‐catenin/TCF‐dependent pro‐survival genes *IGF‐1* and *WISP‐2* were upregulated by Wnt3a alone and with H_2_O_2_. Neutralization of WISP‐2, but not IGF‐1, reduced the anti‐apoptotic effect of Wnt3a, implying a role for WISP‐2 in Wnt3a‐mediated survival. Furthermore, as we demonstrated Wnt3a‐mediated WISP‐2 upregulation was inhibited by β‐catenin/TCF inhibition, we established direct links between Wnt3a, β‐catenin/TCF and WISP‐2.

This is the first report of an anti‐apoptotic role for WISP‐2 in VSMCs. Previous studies revealed WISP‐2 inhibited proliferation, migration, invasion and matrix degradation in rat VSMCs but had no effect on apoptosis (Delmolino, Stearns, & Castellot, 2001; Lake & Castellot, 2003; Lake, Bialik, Walsh, & Castellot, 2003; Myers, Wei, & Castellot, 2014). However, in these studies, only basal apoptosis was investigated in VSMCs treated with serum, which itself a pro‐survival factor (Lake & Castellot, 2003; Lake et al., 2003). Exactly how WISP‐2 promotes VSMC survival is unclear. In other cell types, inhibition of apoptosis by WISP‐2 has been reported alongside Akt and MAPK1 phosphorylation (Chowdhury et al., 2014) or WISP‐2 binding to β_1_ integrin and PI3K/Akt activation (Ohkawa et al., 2011).

This study reports, for the first time, upregulation of Wnt3a and WISP‐2 proteins in atherosclerosis near to macrophages and fibrous cap VSMCs, implying a role for these pro‐survival factors in disease*.* Furthermore, Wnt3a and WISP‐2 co‐localized, which in the light of our in vitro data suggests that Wnt3a may upregulate WISP‐2 expression in plaques. That said that WISP‐2 protein was also detected in Wnt3a negative areas, inferring that WISP‐2 may be upregulated by additional plaque molecules or accumulates in the extracellular space after secretion.

### 3.3 The divergence of Wnt3a and Wnt5a pathways was not due to differential Fzd binding

siRNA was performed to determine whether preferential binding of Wnt3a and Wnt5a to Fzd1 and Fzd6 was responsible for the divergent pro‐survival pathways. However, knockdown revealed that Wnt3a and Wnt5a signalled through both Fzd receptors. To determine the cause of differential signalling, a more complete understanding of the intracellular proteins affected by the Wnts is required.

### 3.4 The pro‐survival effect of Wnt5a, but not Wnt3a, was lost with age

Wnt3a inhibited H_2_O_2_‐induced apoptosis in VSMCs from both young and old mice, whereas Wnt5a‐mediated rescue was lost with age. Similarly, WISP‐1 upregulation by Wnt5a was also blunted with age. Interestingly, a previous study by Marchand et al. (2011) found that Wnt3a‐induced proliferation and cyclin‐D1 expression were lost with age in rat VSMCs, perhaps implying the effect of age on Wnt signalling may be species‐dependent.

### 3.5 LRP5, LRP6 and Dkk3 mRNAs, but not proteins, were altered with age

It was hypothesized that impaired Wnt5a signalling with age may result from altered expression of Wnt signalling components. Although the majority of Wnt signalling components were unaffected by age, expression of *Wnt2, Wnt8a, NCAD*,* LRP5* and *LRP6* mRNAs was significantly reduced in old VSMCs, while *Dkk3* mRNA was significantly increased. Unfortunately, changes in LRP6 and Dkk3 were not observed at the protein level, while LRP5 protein was undetectable. These data suggest that changes in LRP6 and Dkk3 were not responsible for the impaired Wnt5a signalling with age. It is also unlikely that altered expression of LRP5 could cause loss of Wnt5a signalling, as LRP6 appears to be the predominant co‐receptor in VSMCs (Supporting Information Table S3, Wang, Adhikari, Li, & Hall, 2004).

### 3.6 Wnt5a‐mediated activation of CREB was impaired with age

In young and old VSMCs, Wnt5a successfully induced β‐catenin nuclear translocation and similarly affected *AXIN‐2* and *TCF‐7* expression, implying that β‐catenin activation by Wnt5a was unaffected by age. This is in keeping with a report by Marchand *et al*. that described successful Wnt3a‐mediated β‐catenin activation in old rat VSMCs, despite the failure of this Wnt to induce proliferation and cyclin‐D1 expression in aged cells (Marchand et al., 2011). However, we found Wnt5a failed to increase levels of phosphorylated CREB and expression of the CREB‐responsive gene *HAS1* in old VSMCs. These data suggest that impaired CREB activation may underpin the loss of Wnt5a‐mediated survival with age. This conclusion is supported by the finding that Wnt3a‐mediated survival, which we have shown does not involve CREB activation, was unaffected by aging.

### 3.7 Summary

We report that Wnt3a and WISP‐2 are upregulated in human atherosclerosis. In addition, we have shown that in contrast to the pro‐survival pathway used by Wnt5a, Wnt3a‐mediated inhibition of H_2_O_2_‐induced VSMC apoptosis required β‐catenin/TCF signalling and subsequent upregulation of WISP‐2 (Figure 6). This divergence was not due to differential Fzd1 or Fzd6 activation, but does result in a differential effect of aging. Specifically, we showed that although the pro‐survival effect of Wnt3a was unaffected by age, Wnt5a‐mediated inhibition of VSMC apoptosis was lost due to impaired CREB phosphorylation and subsequent WISP‐1 upregulation (Figure 6). These data suggest that aging may underlie the disparity between Wnt5a, WISP‐1 and VSMC survival previously observed in unstable human plaques (Mill et al., 2011, 2014, 2010 ). Finally, these results suggest that restoring Wnt‐mediated CREB activation and WISP‐1 expression in aged VSMCs may provide a novel therapeutic tool to promote plaque stability in elderly patients with atherosclerosis.

## 4 EXPERIMENTAL PROCEDURES

### 4.1 Isolation and culture of VSMCs

Housing, care and all procedures involving mice were performed in accordance with the guidelines and regulations of the University of Bristol and the United Kingdom Home Office (PPL30_3064). The investigation conforms to the *Guide for the Care and Use of Laboratory Animals* published by the US National Institutes of Health (NIH Publication No. 85‐23, revised 1996). For experiments relating to the Wnt3a pro‐survival pathway, primary VSMCs were isolated from explants of aortic tissue from TOPGAL mice, as previously described (Tsaousi et al., 2011). These transgenic mice were originally obtained from Professor Yingzi Yang (Topol et al., 2003) and express a β‐catenin/TCF‐responsive transgene (DasGupta & Fuchs, 1999) although this transgene was not utilized in this study. For experiments investigating aging, primary aortic VSMCs were isolated from 2 month or 18–20 month C57BL6/J mice purchased from Charles River, Margate, UK. In both cases, cells were grown in DMEM supplemented with 10% FBS, 2 mM l‐glutamine, 100 units/ml penicillin, 100 µg/ml streptomycin and 8 µg/ml gentamycin (10% FBS/DMEM). We confirmed that VSMCs from 18–20 month mice had significantly lower levels of klotho mRNA compared to cells from 2 month mice confirming the age of the VSMCs in vitro (0.65 ± 0.07‐fold vs. young, one‐sample *t* test, *p* < 0.05, *n* = 4). VSMCs between passages 2 and 10 were seeded according to the densities in Supporting Information Table S1 and left to adhere overnight at 37°C, 5% CO_2_. VSMCs were then quiesced in serum‐free DMEM (SFM) for 24 hr and stimulated with 400 ng/ml recombinant human Wnt3a protein (5036‐WN; R&D Systems, Oxfordshire, UK) or Wnt5a protein (645‐WN; R&D Systems) in the absence or presence of 100 μM H_2_O_2_ (H1009; Sigma‐Aldrich, Dorset, UK) or with 100 ng/ml recombinant mouse IGF‐1 protein (250–19; PeproTech, London, UK) or 500 ng/ml recombinant human WISP‐2 protein (120‐16, PeproTech). To investigate the role of TCF, two β‐catenin/TCF inhibitors were employed; 1 µM CCT031374 hydrobromide (4675; Tocris Bioscience, Bristol, UK) or 25 µM iCRT‐14 (4299; Tocris Bioscience). For experiments using CCT031374 hydrobromide, the inhibitor was added in combination with Wnt3a ± H_2_O_2;_ however, for experiments using iCRT‐14, an additional one hour pre‐incubation with iCRT‐14 was employed. In both cases, DMSO was used as a vehicle control, 0.002% DMSO was used for CCT031374 hydrobromide or 0.05% DMSO for iCRT‐14. For neutralization experiments, 10 µg/ml of antibodies raised against IGF‐1 (ab9572; Abcam, Cambridge, UK) or WISP‐2 (sc25442, Santa Cruz Biotechnology, Heidelberg, Germany) was also added.

### 4.2 β‐catenin/TCF reporter

To quantify β‐catenin/TCF signalling, VSMCs were transfected with either TOPFlash plasmids (12456; Addgene, Middlesex, UK) or negative control mutated FOPFlash plasmids, in combination with renilla luciferase plasmids to normalize for transfection efficacy (pRL‐TK Vector, E2241; Promega, Southampton, UK). Full protocol is given in the Supporting Information.

### 4.3 Knockdown of WISP‐1, Fzd1 and Fzd6 expression

About 200 pmol of silencing RNAs (siRNAs) for WISP‐1 (S100212702 and S102673370), Fzd1 (SI02674252 and SI00218771) or Fzd6 (SI02666979 and SI02708510; Qiagen, Manchester, UK) was introduced into VSMCs using the method described previously (Mill et al., 2014; Tsaousi et al., 2011). Alternatively, AllStars Negative Control siRNA (1027281; Qiagen) was added. 8 x 10^5^ cells (WISP‐1) or 6 x 10^5^ cells (Fzd1/6) were included in each nucleofection, then VSMCs were seeded at 8 x 10^4^ or 6 x 10^4^ cells/well in 24‐well plates and incubated overnight (WISP‐1) or for 6 hr (Fzd1/6) at 37°C, 5% CO_2_ to adhere. The knockdown efficiency of these protocols has been previously reported (Mill et al., 2014; Tsaousi et al., 2011).

### 4.4 Immunocytochemistry

Immunocytochemistry for CC3 and β‐catenin was performed to analyse cell death after 24‐hr stimulation with Wnt ± H_2_O_2_ or to analyse β‐catenin localization after 30 min. VSMCs were fixed with 3% paraformaldehyde/PBS and permeabilized with 0.1%–0.2% Triton X‐100/PBS. After blocking with 20% goat serum/PBS, 1 µg/ml CC3 antibody (AF835; R&D Systems) in 1% BSA/PBS or 2.5 µg/ml β‐catenin antibody (610154; BD Transduction Laboratories, Oxford, UK) in PBS was added overnight at 4°C. Bound antibodies were detected with biotinylated goat anti‐rabbit IgG (B7389; Sigma‐Aldrich) or biotinylated goat anti‐mouse IgG (BA9200; Vector Laboratories, Peterborough, UK) and then DyLight‐488 Streptavidin (SA‐5488‐1; Vector Laboratories) diluted 1:200 in PBS. Coverslips were mounted in ProLong Gold and DAPI (P36931; Invitrogen, Paisley, UK).

### 4.5 Immunohistochemistry

Human coronary arteries were isolated from cadaveric hearts donated to the Bristol Coronary Artery Biobank under National Research Ethics Service approval (08/H0107/48). Informed consent was given by relatives of the deceased. The investigation conformed to the principles outlined in the Declaration of Helsinki. Donors for clean vessels were aged 11–29 years (*n* = 4), and donors for atherosclerotic vessels were aged 34–63 years (*n* = 11).

Immunohistochemistry was performed for Wnt3a (7 µg/ml, ab28472, Abcam) and WISP‐2 (4 µg/ml, sc25442, Santa Cruz Biotechnology) plus CD68 to identify macrophages (0.8 µg/ml, MO876, Dako, Cambridgeshire, UK) and α‐smooth muscle actin to identify VSMCs (3.1 µg/ml, A2547, Sigma‐Aldrich) (full method in Supporting Information**). Corresponding concentrations of the appropriate non‐immune IgGs were used as negative controls. The intensity of Wnt3a and WISP‐2 staining was scored from 1 to 4 by eye (1 representing low levels and 4 representing the highest level of staining observed overall), and co‐localization with macrophages and VSMCs was examined visually. Results from a single observer are presented in this paper. However, to increase the robustness of the scoring system, the staining was also scored by a second independent observer. The inter‐observer reliability of this scoring, calculated by a Spearman’s rank correlation, was significantly correlated (Wnt3a *p* = 0.0021, WISP‐2 *p* < 0.0001), thus supporting the findings of the first observer.

### 4.6 Quantitative PCR

RNA was analysed from quiesced VSMCs or cells stimulated with Wnt ± H_2_O_2_ for 4 hr. Details of RNA extraction, cDNA synthesis and 384‐well and 96‐well QPCR are given in the Supporting Information**. Briefly, 384‐well TaqMan Array Micro‐Fluidic Cards were designed and purchased from Applied Biosystems, whereas 96‐well QPCR was performed using the primers detailed in Supporting Information Table S2. mRNA levels were normalized as detailed in the legend.

### 4.7 Western blotting

To investigate changes in protein expression, VSMCs were incubated with Wnt ± H_2_O_2_ for 24 hr. Proteins were extracted by 5% SDS lysis, and total protein concentration was determined by Micro Bicinchoninic Acid Protein Assay Kit (23235; Thermo Fisher Scientific, Massachusetts, USA). Western blots were performed as previously described (Uglow et al., 2003) using 1.6 µg/ml phosphorylated CREB (ser133) antibody (9196; Cell Signalling Technologies, Hertfordshire, UK) diluted in 5% milk/TBST overnight at 4ºC or 0.54 µg/ml LRP6 (3395; Cell Signalling Technologies) or 1 µg/ml Dkk3 (sc14959, Santa Cruz Biotechnology) antibodies diluted in 5% BSA/TBST overnight at 4ºC. Levels of each protein (optical density [O.D.] x mm^2^) were normalized to the corresponding β‐actin (110 ng/ml β‐actin antibody, A5316; Sigma‐Aldrich) or stain‐free band (456‐1084; BIO‐RAD, Hertfordshire, UK).

### 4.8 Statistics

Results are expressed as mean ± *SEM*. Where *N* was sufficient, normal distribution was tested by the Kolmogorov and Smirnov test for normality. The following tests were then used; a one‐sample *t* test for data presented as a fold change compared to 1, a Student *t* test for comparing the means of two groups and an ANOVA with a Student–Newman–Keuls multiple comparisons post hoc test for comparing means of more than two groups. Paired or unpaired analysis was used as appropriate. An output of *p* < 0.05 was accepted as significantly different in all tests.

## AUTHOR CONTRIBUTIONS

BAB was responsible for the acquisition, analysis and interpretation of most of the data presented. GMC optimized Wnt3a immunohistochemistry and helped analysis and interpretation of staining. CEJM acquired, analysed and interpreted data showing Wnt5a‐mediated survival with age and was responsible for optimizing many protocols. HW helped with data acquisition and interpretation by sacrificing mice, helping to isolate VSMCs, optimizing protocols and providing advice. GDA helped with steering project direction. JLJ helped with design of the work, data interpretation and steering project direction. SJG was responsible for the conception and design of the work, data interpretation and steering project direction. These authors have been involved in drafting or revising this paper and have given approval for publication and accountability for this work.
