# Supplementary material for: Aging differentially modulates the Wnt pro‐survival signalling pathways in vascular smooth muscle cells
Source: Aging Cell. 2018 Dec 12;18(1):e12844. doi: 10.1111/acel.12844 (PMC6351844; doi:10.1111/acel.12844)
Supplement: Supplementary file 1 [file ACEL-18-e12844-s001.pdf]

Supporting Information

Methods

β-catenin/TCF reporter

To quantify β-catenin/TCF signalling, 2.5µg M50 Super 8x TOPFlash plasmids (12456, ADDGENE, Middlesex, UK), containing 7 TCF/LEF binding sites controlling firefly luciferase gene expression, were introduced into 8x10^5 VSMCs using the Amaxa Basic Nucleofector kit for primary smooth muscle cells (VP1-1004, LONZA, Verviers, Belgium). 2.5µg M51 Super 8x FOPFlash plasmids, containing 6 mutated TCF/LEF binding sites, were used as a negative control (12457, ADDGENE, Middlesex, UK). Alongside TOPFlash or FOPFlash plasmids, 0.5µg renilla luciferase plasmids were introduced to normalise for transfection efficacy (pRL-TK Vector, E2241, PROMEGA, Southampton, UK). Following nucleofection using program A-033 on the AMAXA Nucleofector II (AAd-1001N), VSMCs were seeded at 2x10^5 cells/well in 12-well plates and incubated overnight at 37°C, 5% CO_2. VSMCs were then quiesced for 24 hours then treated with Wnt±H_2O_2 for 8 hours. Firefly and renilla luciferase activity was quantified using the Dual-Luciferase Reporter Assay System (E1910, PROMEGA, Southampton, UK). Luminescence was quantified for two separate 1 second exposures using the Promega Glomax 96 microplate luminometer. The firefly luciferase measurements were normalised to renilla luciferase activity and supernatant protein concentration as measured by the Micro Bicinchoninic Acid Protein Assay Kit (23235, THERMO FISHER SCIENTIFIC, Massachusetts, USA).

Immunohistochemistry

Paraffin-wax sections (3µm) were de-waxed by three consecutive 5 minute washes in 100% (v/v) clearene, and rehydrated by two consecutive 5 minute washes in 100% (v/v) alcohol, followed by a single 5 minute wash in 70% (v/v) alcohol and then distilled water. To inhibit endogenous peroxidase activity slides were incubated in 0.3% (v/v) H_2O_2 for 7 minutes at room temperature. Next antigen retrieval was achieved by submersing slides in 10mM citrate buffer (pH 6.0) and microwaving twice at 800W for 5 minutes. Slides were left to cool for 30 minutes at room temperature and then cold tap water was gradually poured over the slides to dilute the citrate buffer. The slides were then washed three times in PBS and sections were delineated using a hydrophobic pen. To block non-specific binding, sections were incubated with 20% (v/v) goat serum in PBS for 30 minutes at room temperature. The goat serum was removed and primary antibodies for Wnt3a (7µg/ml, ab28472, ABCAM, Cambridge, UK), WISP-2 (4µg/ml, sc25442, SANTA CRUZ BIOTECHNOLOGY, Heidelberg, Germany), CD68 (0.8µg/ml, MO876, DAKO, Cambridgeshire, UK) and α-smooth muscle actin (3.1µg/ml, A2547, SIGMA ALDRICH, Dorset, UK) diluted in 1% (w/v) BSA/PBS were added and incubated overnight at 4^{0}C. Corresponding concentrations of the appropriate non-immune IgGs were used as negative controls. The following

day, slides were washed three times in PBS then incubated with biotinylated secondary antibodies (either biotinylated goat-anti-mouse IgG (BA-9200, VECTOR LABORATORIES, Peterborough, UK) or goat-anti-rabbit IgG (B7389, SIGMA ALDRICH, Dorset, UK) as appropriate) diluted 1:200 in 1% (w/v) BSA/PBS for 45 minutes at room temperature. Sections were then washed three times in PBS then treated with ExtrAvidin-peroxidase (E2886, SIGMA ALDRICH, Dorset, UK) diluted 1:200 in 1% (w/v) BSA/PBS for 45 minutes at room temperature. Sections were then washed three times in PBS and incubated with SigmaFast 3,3'-diaminobenzidine (DAB: D4293, SIGMA ALDRICH, Dorset, UK) for 3-10 minutes at room temperature. Sections were then washed in distilled water and stained with the nuclear stain Mayer's hematoxylin for 30 seconds. Slides were washed with tap water to 'blue' the hematoxylin until the water appeared clear. Sections were then dehydrated by one 5 minute wash in 70% (v/v) alcohol, two 5 minute washes in 100% (v/v) alcohol and then three 5 minute washes in 100% (v/v) clearene. Slides were then mounted in DPX mounting medium (10050080, THERMO FISHER SCIENTIFIC, Massachusetts, USA) and cured overnight at room temperature. The following day the sections were imaged using an Olympus BX41 microscope and Q-capture pro 6.0 software.

Quantitative PCR

To investigate changes in mRNA expression with age, young and old VSMCs were quiesced for 48 hours. RNA was extracted from VSMCs and purified using the PureLink RNA mini-kit (12183025, AMBION, Paisley, UK). 500ng of RNA and 4µL of SuperScript VILO Master Mix (11755-050, INVITROGEN, Paisley, UK) was then added to RNase-free PCR tubes and the samples were made up to 20µL with RNase-free water. The samples were heated to 25°C for 10 minutes, then 42°C for 90 minutes and finally 85°C for 5 minutes (C1000™ thermocycler, BIO-RAD, Dorset, UK). The resultant cDNA was then cooled to 4˚C. To enable simultaneous analysis of 48 Wnt related genes, 384-well Taqman Array Micro-Fluidic Cards were designed and purchased from Applied Biosystems. 20µL of cDNA, 35µL of nuclease-free water and 55µL of Taqman Universal Master Mix-II with UNG (4440042, APPLIED BIOSYSTEMS, Paisley, UK) were mixed, then 100µL/sample was added to each reservoir of the array cards. Cards were centrifuged twice at 1200rpm for 1 minute to evenly distribute the cDNA between two rows of the array card and cards were sealed. Comparative C_T was then analysed using the ViiA 7 Real-Time PCR System and ViiA 7 RIO software. The card layout and thermocycling conditions were imported using the Gene expression micro-fluidic card CD (4480358, APPLIED BIOSYSTEMS, Paisley, UK) provided with the array cards.

For genes that were not included on the array plate, or to analyse the effect of Wnt on mRNA expression, 96-well QPCR was used. cDNA was synthesised using the Transcriptor First Strand cDNA Synthesis Kit (04896866001, ROCHE, West Sussex, UK) and QPCR was performed using the LightCycler 480 SYBR Green I Master kit (04707516001, ROCHE, West Sussex, UK) and LightCycler 480 II (ROCHE, West Sussex, UK). Primer sequences are detailed in Supplementary Table 2. mRNA levels

were normalised to *18s* mRNA, except for *AXIN-2, IGF-1, TCF-7* and *WISP-2* which were normalised to *36B4* mRNA, and *WISP-1* which was quantified from a standard curve.

Supplementary Table 1: Seeding densities for vascular smooth muscle cells.

Protocol	Plate type	Seeding density
Quantitative-PCR	6-well plate	1 x 10^5 cells/well
Western blotting	12-well plate	8 x 10^4 cells/well
TOPFlash reporter assay	12-well plate	2 x 10^5 cells/well
Immunocytochemistry for cleaved-caspase 3 or β-catenin	24-well plate (containing coverslips)	4 x 10^4 cells/well
Immunocytochemistry for cleaved-caspase 3 following silencing RNA nucleofection	24-well plate (containing coverslips)	8 x 10^4 cells/well

Supplementary Table 2: Primer sequences or catalogue numbers.

Gene	Primer Sequence	Working Concentration	Company	Catalogue number
18s	F: 5’-CGCGGTTCTATTTTGTTGGT<br>R: 5’-CTTCAAACCTCCGACTTTCG	2µM	Sigma Aldrich	N/A
36B4	F: 5’-GCCCAGGGAAGACAGGGGA<br>R: 5’-GCGCATCATGGTGTTCTTGCCCA	1µM	Sigma Aldrich	N/A
AXIN-2	Not disclosed by the manufacturer	1µM	Qiagen	QT00126539
HAS1	F: 5’- TACTGGGTAGGTGTGAGGAGGCTG<br>R: 5’- GTCCCCAGAAAGCCATGACTCCAG	1µM	Sigma Aldrich	N/A
IGF-1	Not disclosed by the manufacturer	1µM	Qiagen	QT00154469
LRP5	Not disclosed by the manufacturer	1µM	Qiagen	QT01755159
LRP6	Not disclosed by the manufacturer	1µM	Qiagen	QT00164199
sFRP1	Not disclosed by the manufacturer	1µM	Qiagen	QT00167153
sFRP2	Not disclosed by the manufacturer	1µM	Qiagen	QT00101759
sFRP3	F: 5'-CAAGGGACACCGTCAATCTT<br>R:5'-CGATCCTTCCACTTCTCAGC	1µM	Sigma Aldrich	N/A
sFRP4	Not disclosed by the manufacturer	1µM	Qiagen	QT00120491
sFRP5	F: 5'-TCTTCCTCTGCTCGCTCTTC<br>R: 5'-GGGCACAGATCTTGGTCACT	1µM	Sigma Aldrich	N/A
TCF-7	Not disclosed by the manufacturer	1µM	Qiagen	QT01038247
WISP-1	F: 5’-CGTGGAGCAACGGTATGAG<br>R: 5’-GAGAGTGAAGTTCGTGGCC	1µM	Sigma Aldrich	N/A
WISP-2	Not disclosed by the manufacturer	1µM	Qiagen	Q701061571
WIF-1	Not disclosed by the manufacturer	1µM	Qiagen	QT01065848

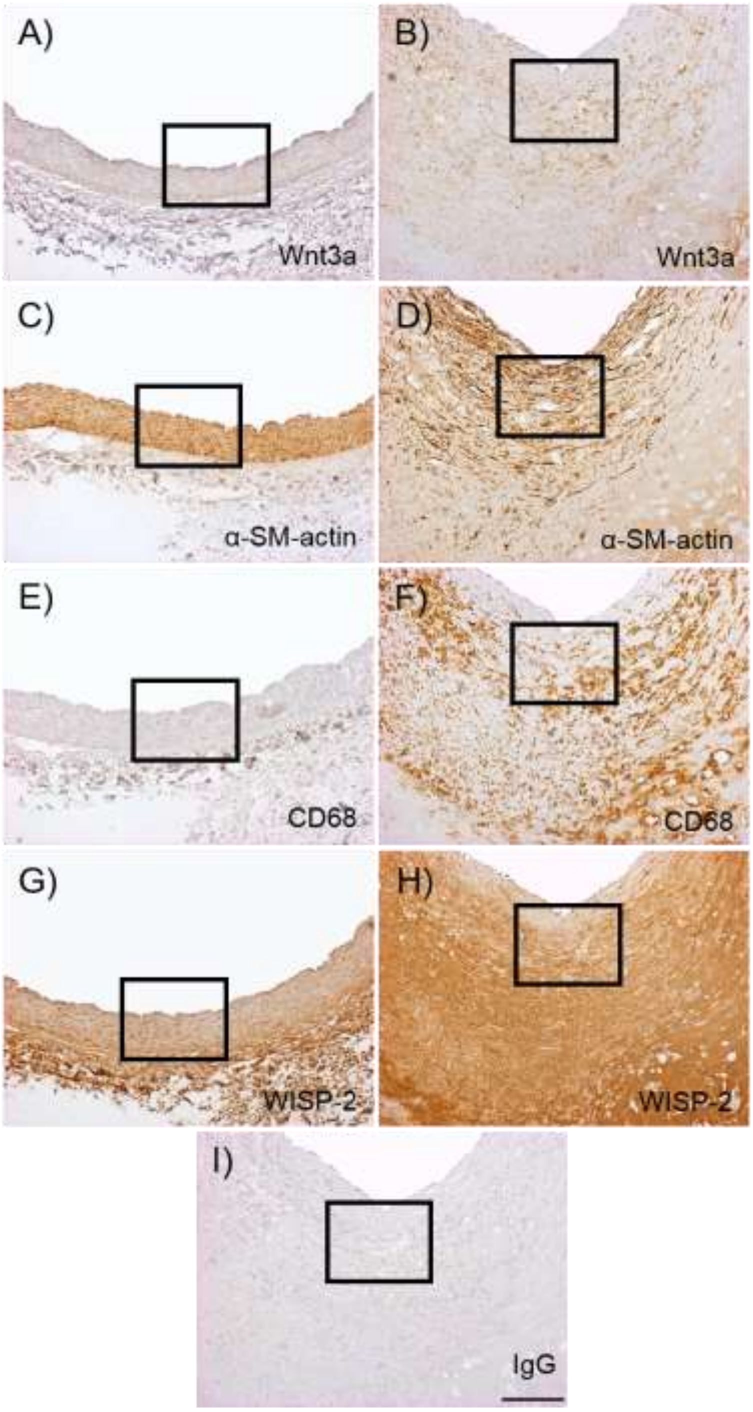

Supplementary Figure 1: Wnt3a and WISP-2 proteins are upregulated in human atherosclerotic plaques.

Representative images of immunohistochemistry for Wnt3a (A & B), α-smooth muscle actin (C & D), CD68 (E & F) and WISP-2 proteins (G & H) in control (A, C, E & G) and atherosclerotic (B, D, F & H) human coronary arteries. Non-immune rabbit IgG was included as a negative control for Wnt3a and WISP-2 antibodies (I). The scale bar represents 250μm and applies to all images. The black boxes indicate the location of the higher magnification images shown in Figure 1 of the manuscript.

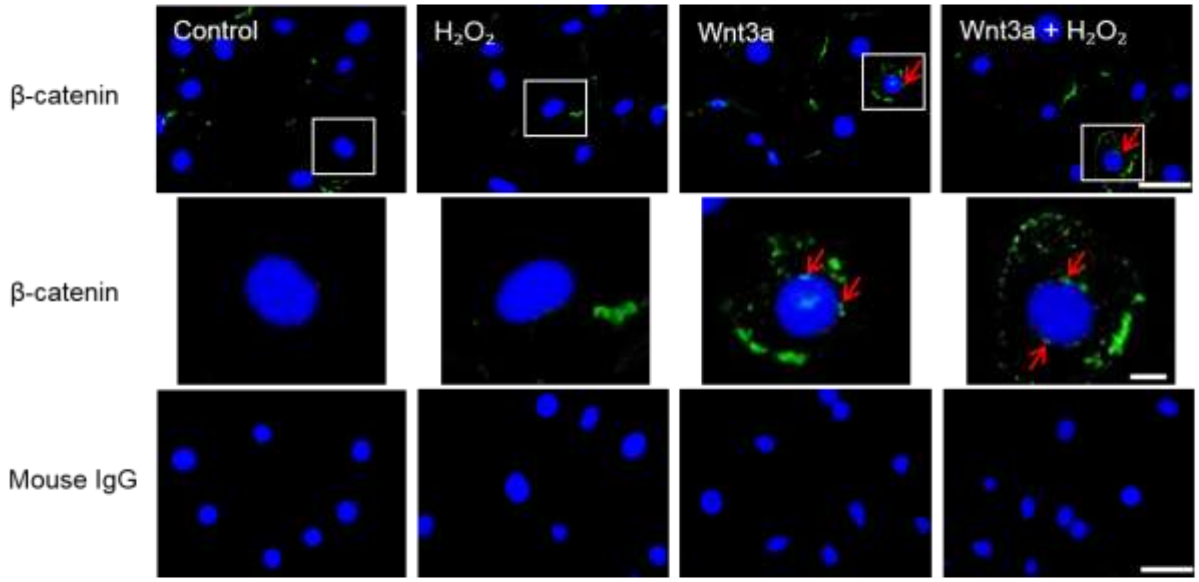

Supplementary Figure 2: Wnt3a protein induced nuclear translocation of β-catenin in the absence and presence of H_2O_2.

Representative images of β-catenin immunofluorescence on young TOPGAL mouse aortic VSMCs stimulated with 100μM H_2O_2, in the presence or absence of 400ng/mL recombinant Wnt3a protein, for 30 minutes. Non-immune mouse IgG was used as a negative control. Red arrows indicate β-catenin nuclear translocation, defined as perinuclear β-catenin staining (green). Blue nuclei: DAPI. For the top and bottom row the scale bar represents 25μm, for the middle row the scale bar represents 5μm.

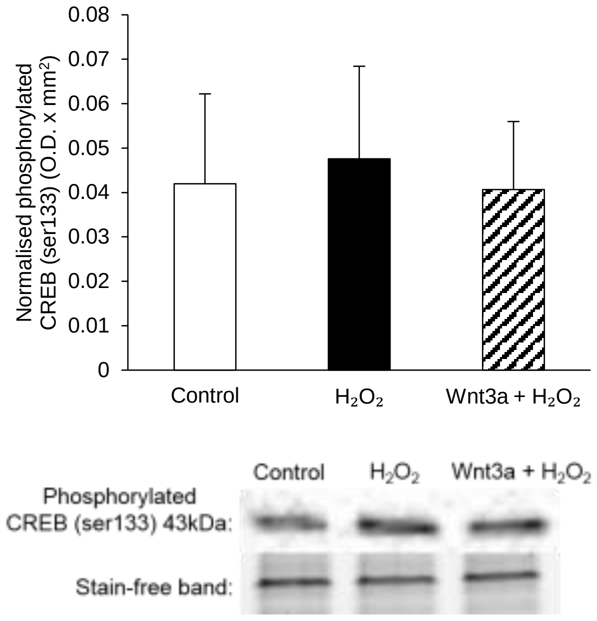

Supplementary Figure 3: Wnt3a protein, in the presence of H_2O_2, did not affect levels of phosphorylated CREB (ser133).

Phosphorylated CREB (ser133) was detected by Western blotting of young TOPGAL mouse aortic VSMCs stimulated with 100µM H_2O_2, in the presence or absence of 400ng/mL recombinant Wnt3a protein, for 10 minutes. Loading was assessed using Bio-Rad stain-free technology and the ChemiDoc MP System. Levels of phosphorylated CREB (ser133) were normalised to the corresponding stain-free band. No significant differences were observed. Repeated measures ANOVA and Student Newman-Keuls post hoc test, n=5. A representative Western blot is shown.

A)

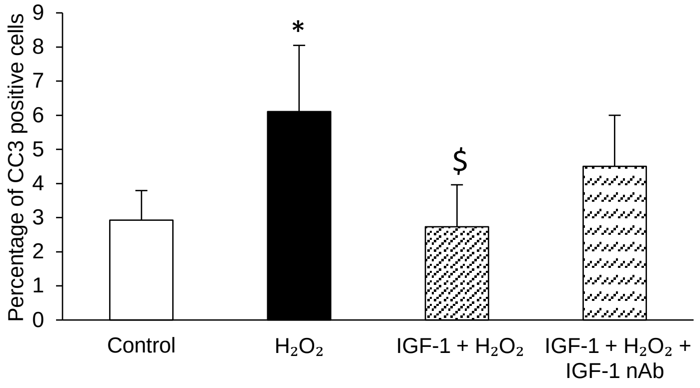

B)

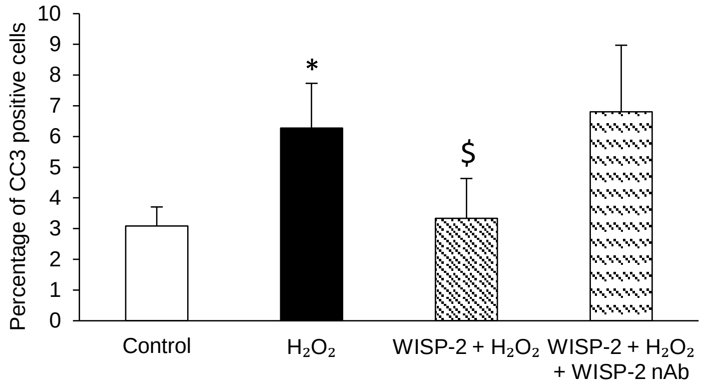

Supplementary Figure 4: WISP-2 and IGF-1 protein rescued VSMCs from H_2O_2-induced apoptosis, which could be inhibited by neutralising antibodies.

Apoptosis was quantified in young TOPGAL mouse aortic VSMCs stimulated with 100µM H_2O_2, in the absence and presence of 100ng/mL recombinant IGF-1 protein and 10µg/mL of IGF-1 neutralising antibody (nAb) (A) or in the absence and presence of 500ng/mL recombinant WISP-2 protein and 10µg/mL of WISP-2 nAb (B), for 24 hours using CC3 immunofluorescence. The number of CC3 positive cells were counted and expressed as a percentage of the total number of cells viewed. * indicates $p<0.05$ vs. control, $ indicates $p<0.05$ vs. H_2O_2, repeated measures ANOVA and Student Newman Keuls post hoc test, n=5.

A)

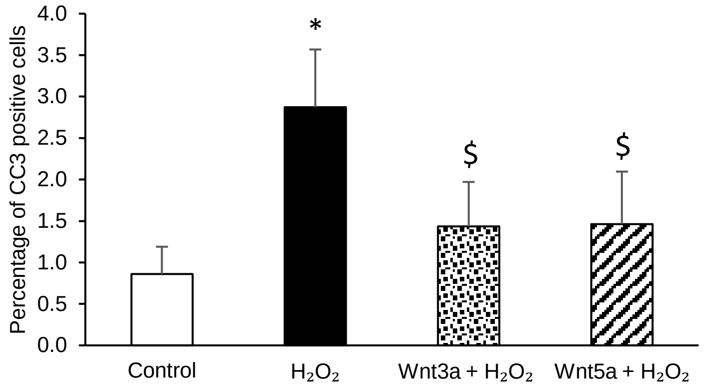

B)

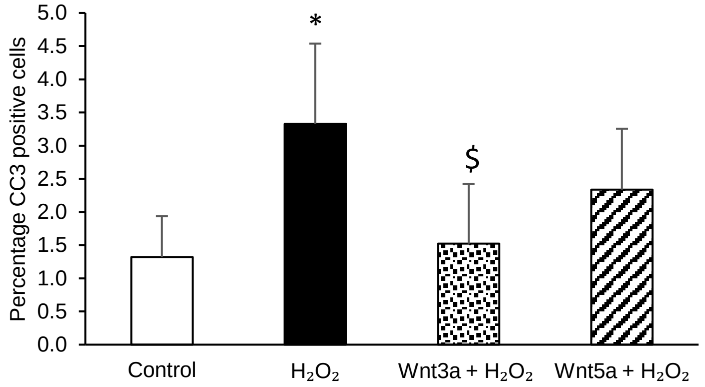

C)

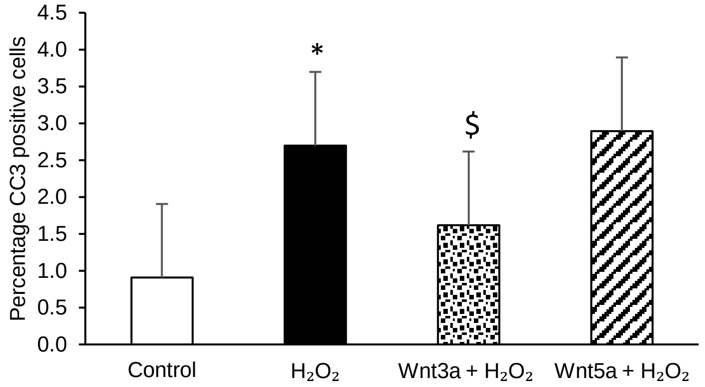

Supplementary Figure 5: The divergent signalling pathways induced by Wnt3a and Wnt5a cannot be explained by the use of different Fzd receptors.

Apoptosis was quantified in young TOPGAL mouse VSMCs transfected with either Allstars negative control siRNA (A) or Fzd1 siRNA (B) or Fzd6 siRNA (C). Six hours after transfection, VSMCs were quiesced in serum free conditions overnight, then 24 hours after transfection cells were stimulated with 100μM H_2O_2, in the presence or absence of 400ng/mL recombinant Wnt3a protein, for a further 24 hours. Apoptosis was quantified using CC3 immunofluorescence. The number of CC3 positive cells were counted and expressed as a percentage of the total number of cells viewed. Error bars represent SEM. * indicates $p<0.05$ vs. control, $ indicates $p<0.05$ vs. H_2O_2, repeated measures ANOVA and Student Newman-Keuls post hoc test, n=7.

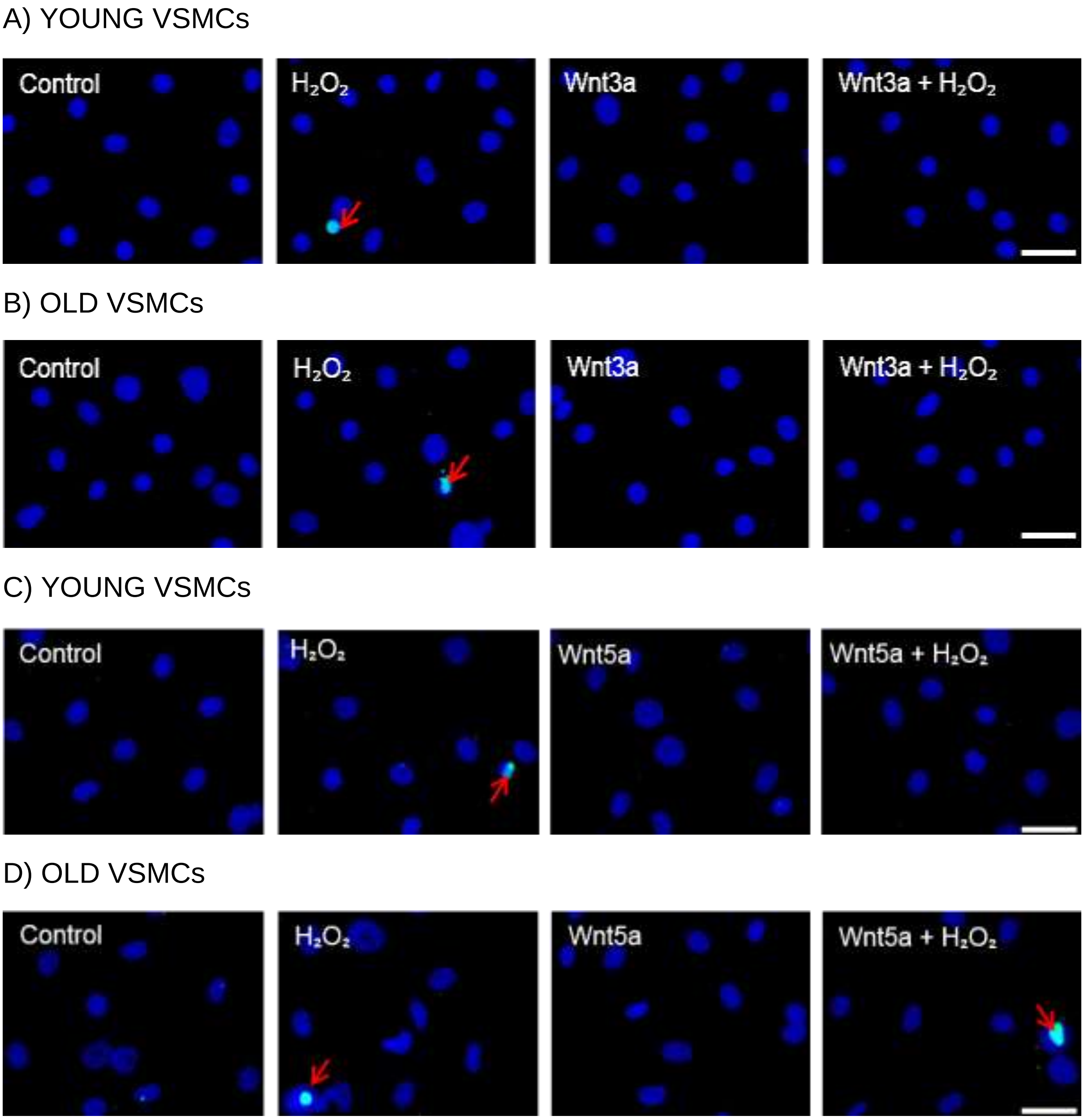

Supplementary Figure 6: Wnt3a protein inhibited H_2O_2-induced apoptosis in VSMCs from young and old mice, whereas Wnt5a-mediated rescue was lost with age.

Apoptosis was quantified in aortic VSMCs isolated from young (A, C) and old mice (B, D) and stimulated with 100µM H_2O_2, in the presence or absence of 400ng/mL recombinant Wnt3a (A, B) Wnt5a protein (C, D) for 24 hours using cleaved caspase-3 (CC3) immunofluorescence. Representative images are shown, red arrows indicate positive cells (green). Blue nuclei:DAPI. The scale bar represents 25µm and applies to all images.

Supplementary Table 3: Changes in mRNA expression of Wnt signalling components with age.

mRNA expression of Wnt signalling components was analysed in aortic VSMCs isolated from young and old mice and quiesced for 48 hours. Genes investigated included Wnt ligands, frizzled receptors (Fzd), low density lipoprotein receptor related protein (LRP) co-receptors, N-cadherin (NCAD) and Wnt inhibitors secreted frizzled-related proteins (sFRPs), Dickkopf (Dkk) proteins and Wnt-inhibitory factor-1 (WIF-1). For each gene, the average C_T value generated from VSMCs from young mice and the fold change in gene expression in cells from old mice is shown. All genes were normalised to *18s* mRNA levels. Blue highlighting indicates mRNAs that were significantly reduced with age, whereas pink highlighting indicates one mRNA that was significantly increased with age ($p<0.05$ vs. young, one sample t-test, n=3-5). ND represents not detected, NS represents non-significant.

	Young C_T value	Old fold change	Significantly different from young?
Wnt1	34.71±0.73	ND	NS
Wnt2	26.73±0.36	0.76±0.05	p<0.05
Wnt2b	26.11±0.44	0.43±0.17	NS
Wnt3	32.43±0.11	1.43±0.56	NS
Wnt3a	ND	ND	NS
Wnt4	29.45±0.06	0.93±0.18	NS
Wnt5a	24.71±0.43	1.24±0.08	NS
Wnt5b	26.71±0.34	0.91±0.13	NS
Wnt6	30.83±0.59	1.18±0.11	NS
Wnt7a	33.61±0.46	2.77±0.97	NS
Wnt7b	32.10±0.25	0.92±0.25	NS
Wnt8a	32.66±0.47	0.48±0.07	p<0.05
Wnt8b	31.87±0.28	2.03±0.47	NS
Wnt9a	26.44±0.12	0.85±0.31	NS
Wnt9b	ND	ND	NS
Wnt10a	29.57±0.31	0.71±0.15	NS
Wnt10b	30.27±0.18	1.20±0.33	NS
Wnt11	28.39±0.33	0.99±0.17	NS
Wnt16	28.84±0.24	1.19±0.46	NS
Fzd1	22.16±0.34	1.12±0.05	NS
Fzd2	23.71±0.23	0.90±0.11	NS
Fzd3	26.87±0.39	1.22±0.09	NS
Fzd4	22.34±0.35	0.99±0.08	NS
Fzd5	23.70±0.28	1.09±0.16	NS
Fzd6	24.31±0.04	0.72±0.24	NS
Fzd7	21.59±0.42	1.05±0.14	NS
Fzd8	23.34±0.22	1.12±0.02	NS
Fzd9	27.84±0.14	0.78±0.16	NS
Fzd10	26.66±0.13	0.79±0.08	NS
LRP5	23.34±0.18	0.80±0.01	p<0.05
LRP6	22.47±0.26	0.73±0.05	p<0.05

Dkk1	34.63±0.56	ND	NS
Dkk2	26.41±0.26	0.80±0.22	NS
Dkk3	19.98±0.51	1.26±0.06	p<0.05
Dkk4	ND	ND	NS
WIF-1	ND	ND	NS
sFRP1	25.87±0.48	0.89±0.36	NS
sFRP2	27.93±0.37	0.99±0.06	NS
sFRP3	24.37±0.85	10.0±3.43	NS
sFRP4	22.12±0.17	0.91±0.28	NS
sFRP5	ND	ND	NS
Ctnnb1 (β-catenin)	19.66±0.38	1.00±0.08	NS
AXIN-2	28.12±0.16	1.13±0.30	NS
NCAD	22.72±0.18	0.57±0.06	p<0.05

A)

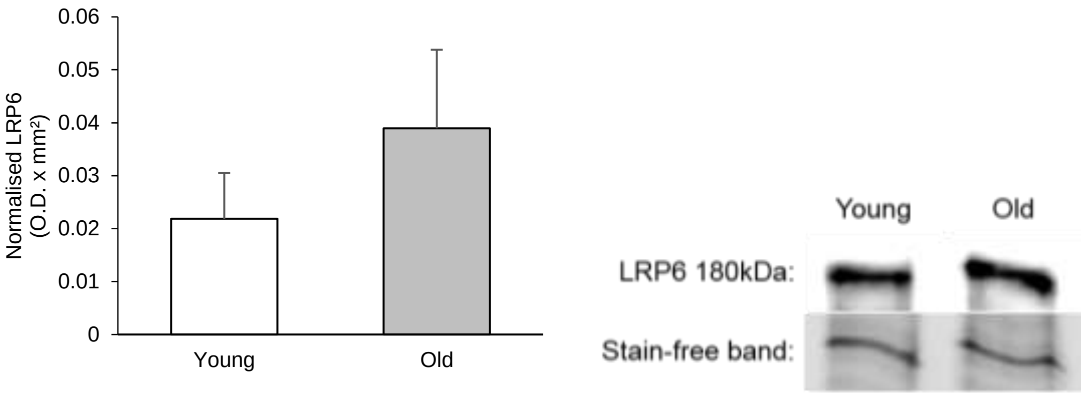

B)

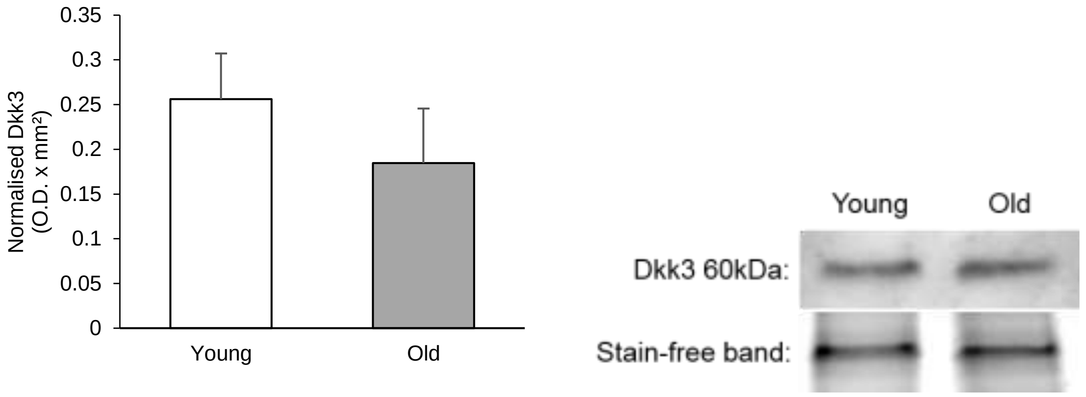

Supplementary Figure 7: LRP6 and Dkk3 protein levels were unaffected by age.

Low density lipoprotein receptor related protein-6 (LRP6) protein (A) and Dickkopf-3 (Dkk3) protein (B) levels were detected by Western blotting of aortic VSMCs isolated from young or old mice. Loading was assessed using Bio-Rad stain-free technology and the ChemiDoc MP System. Levels of LRP6 or Dkk3 were normalised to the corresponding stain-free band. No significant differences were seen with age, unpaired Student’s t-test, n=3-4. Representative Western blots are shown next to each chart.

A) YOUNG VSMCs

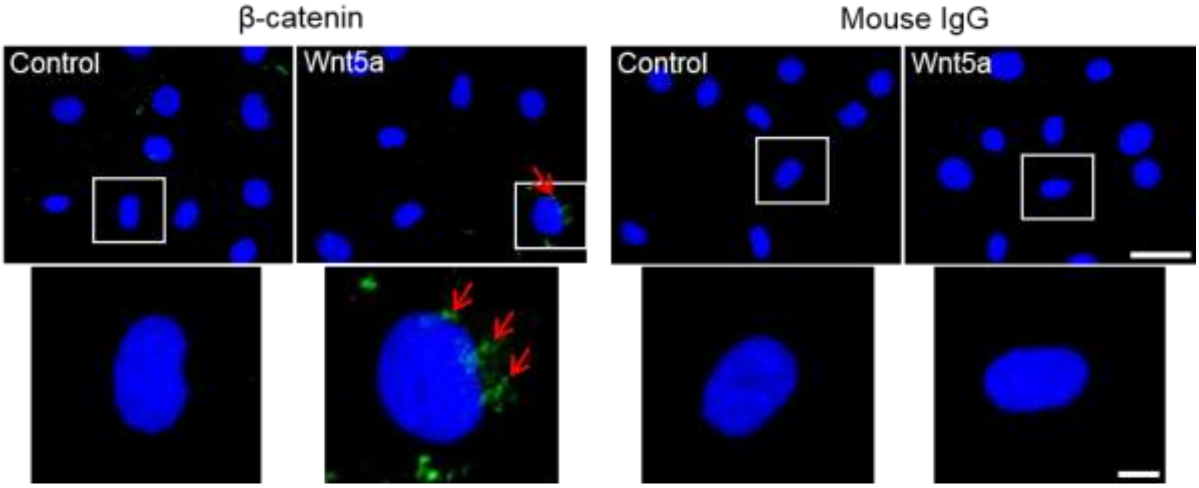

B) OLD VSMCs

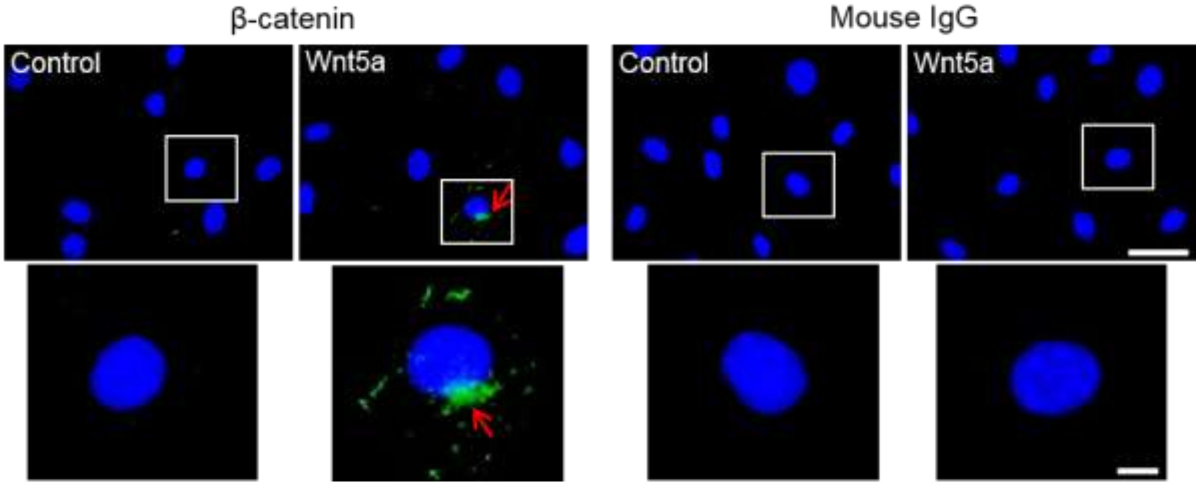

Supplementary Figure 8: Wnt5a-mediated nuclear translocation of β-catenin was unaffected by age.

Representative images of β-catenin immunofluorescence on aortic VSMCs isolated from young (A) and old (B) mice and stimulated with 400ng/mL recombinant Wnt5a protein for 30 minutes. Non-immune mouse IgG was used as a negative control. Red arrows indicate β-catenin nuclear translocation, defined as perinuclear β-catenin staining (green). Blue nuclei: DAPI. The scale bar represents 25µm on the top rows, and 5µm on the bottom rows.

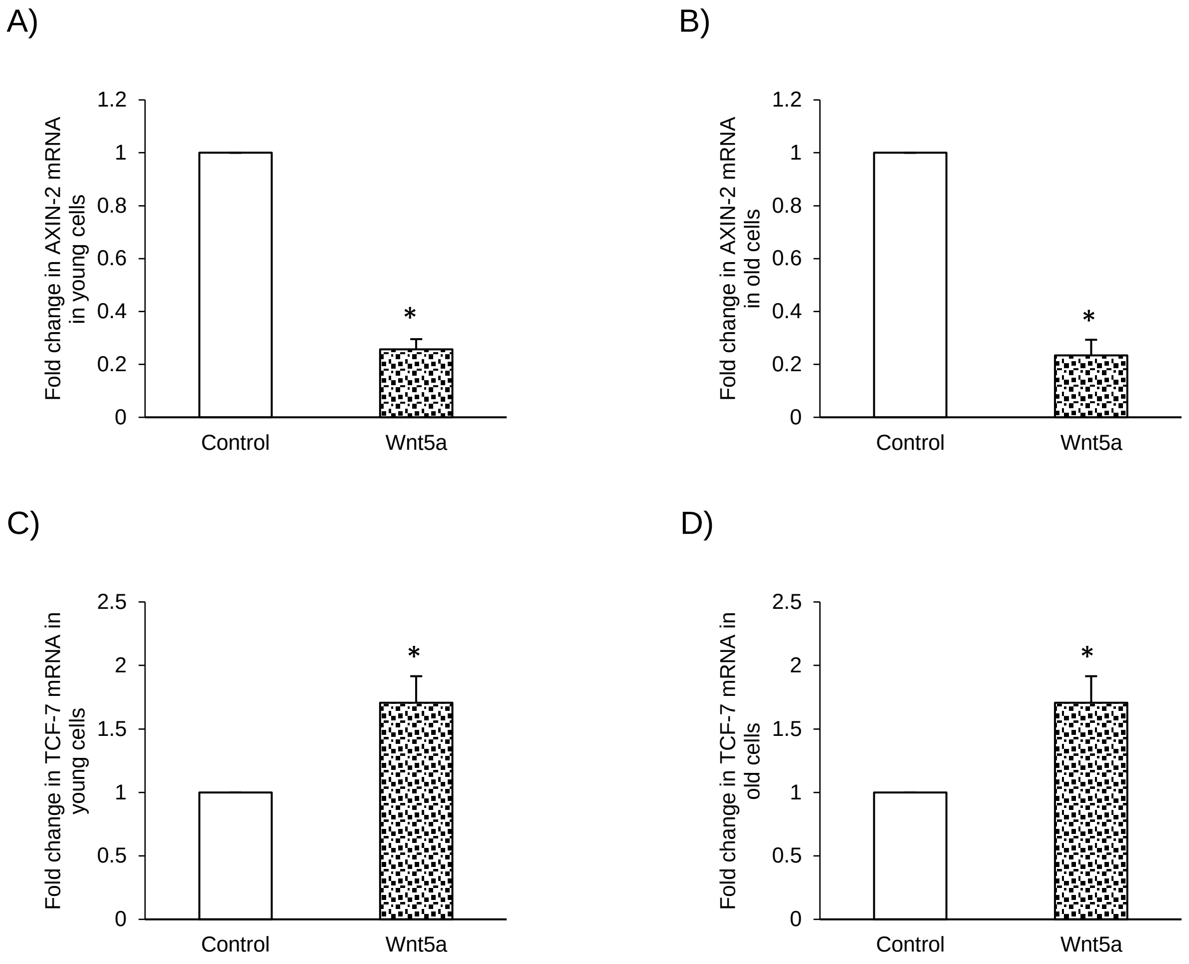

Supplementary Figure **9: Wnt5a affects AXIN-2 and TCF-7 expression similarly in VSMCs from young and old mice.**

AXIN-2 (A & B) and *T-cell factor-7 (TCF-7)* (C & D) mRNA was quantified by QPCR in aortic VSMCs isolated from young (A & C) and old (B & D) mice and stimulated with 400ng/mL recombinant Wnt5a protein for 4 hours. *AXIN-2* or *TCF-7* mRNA levels were normalised to *36B4* mRNA levels. Results are shown as the fold change from control. * indicates $p<0.05$ vs. control, one sample t-test, n=3.
